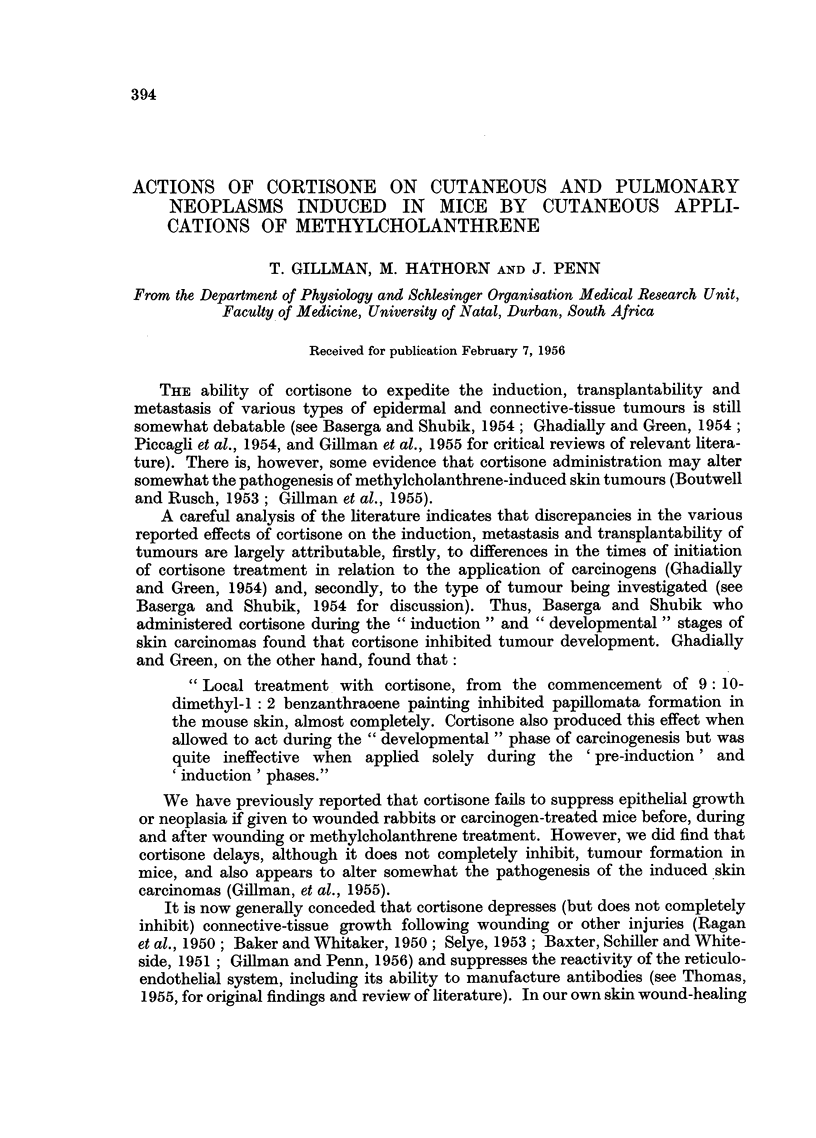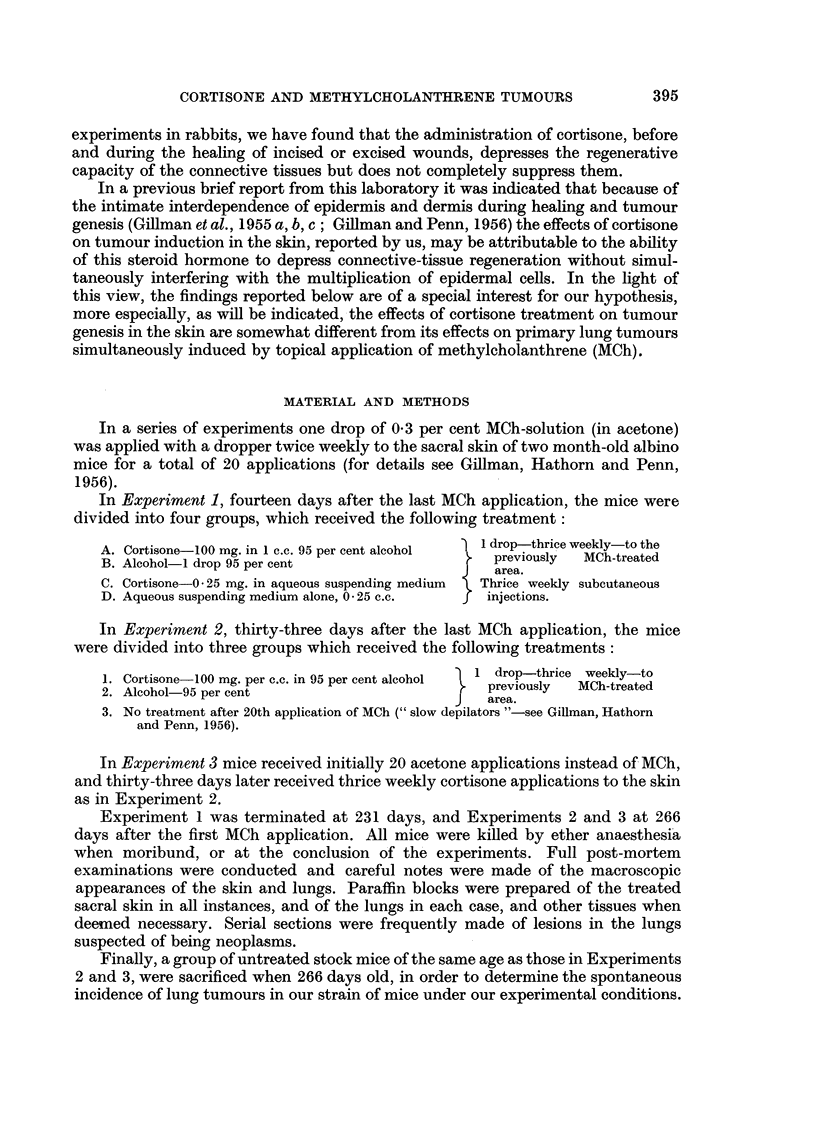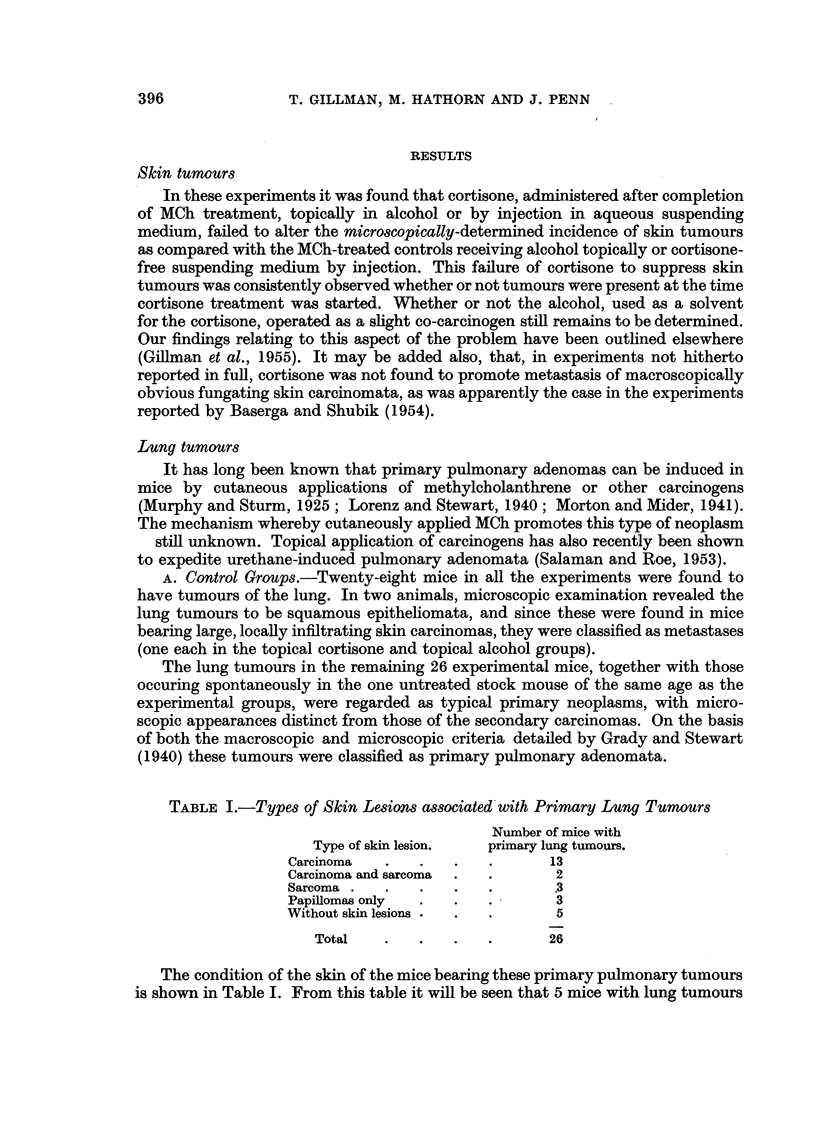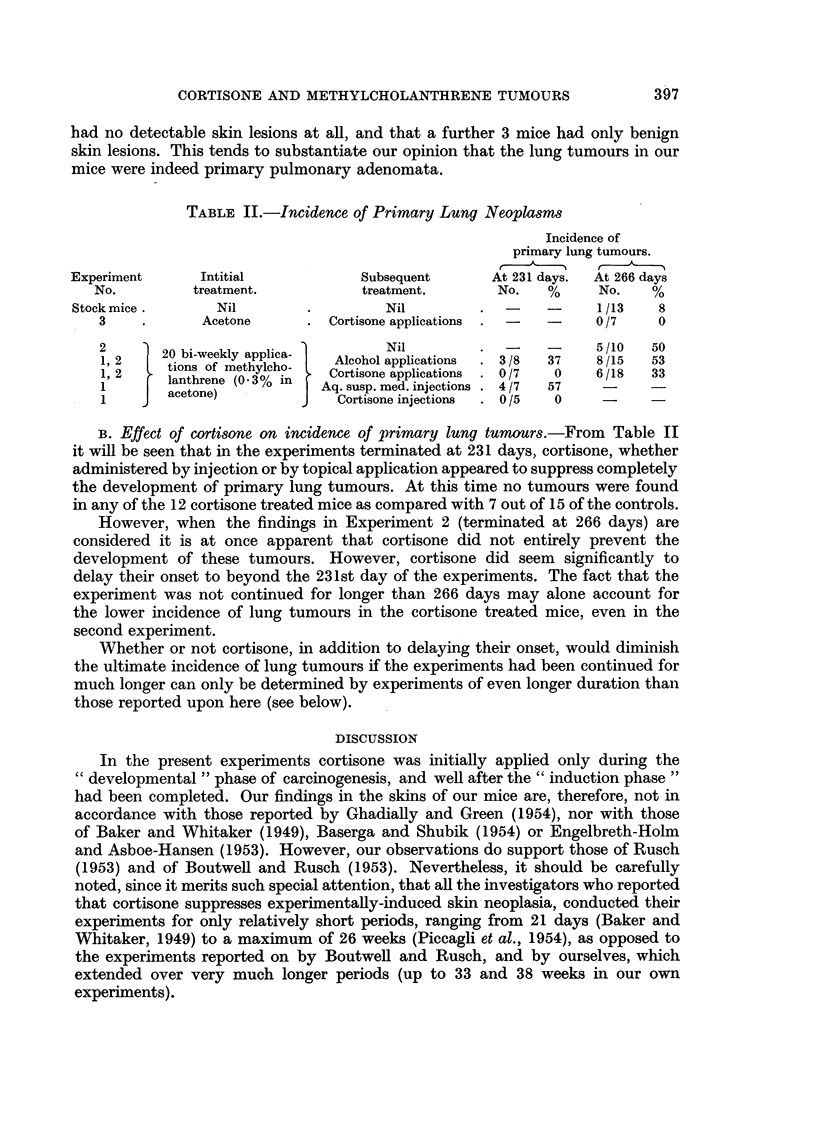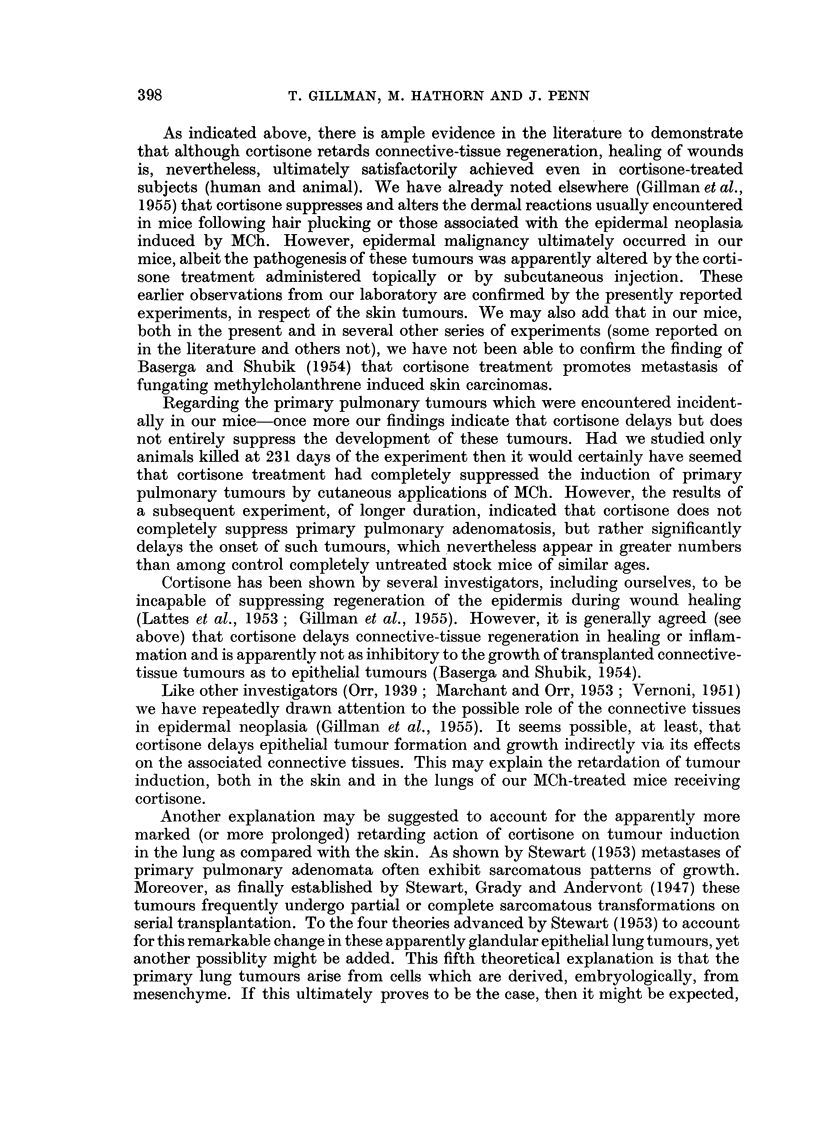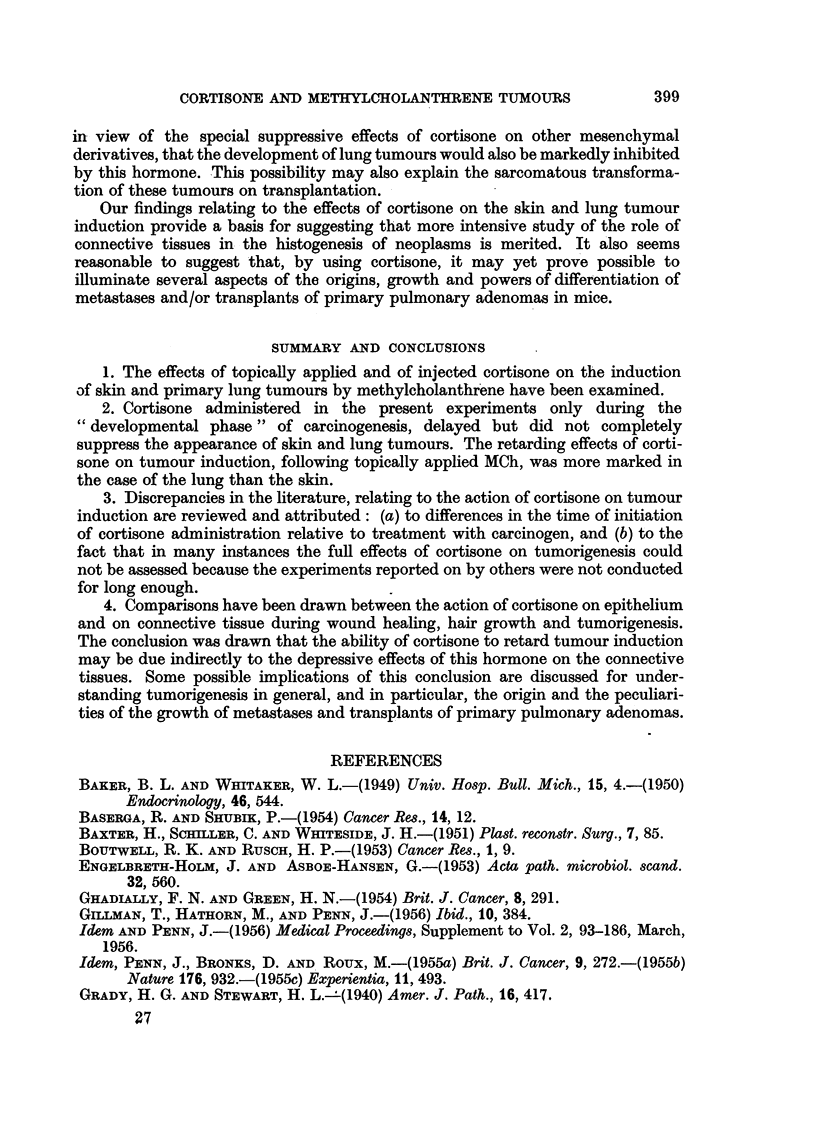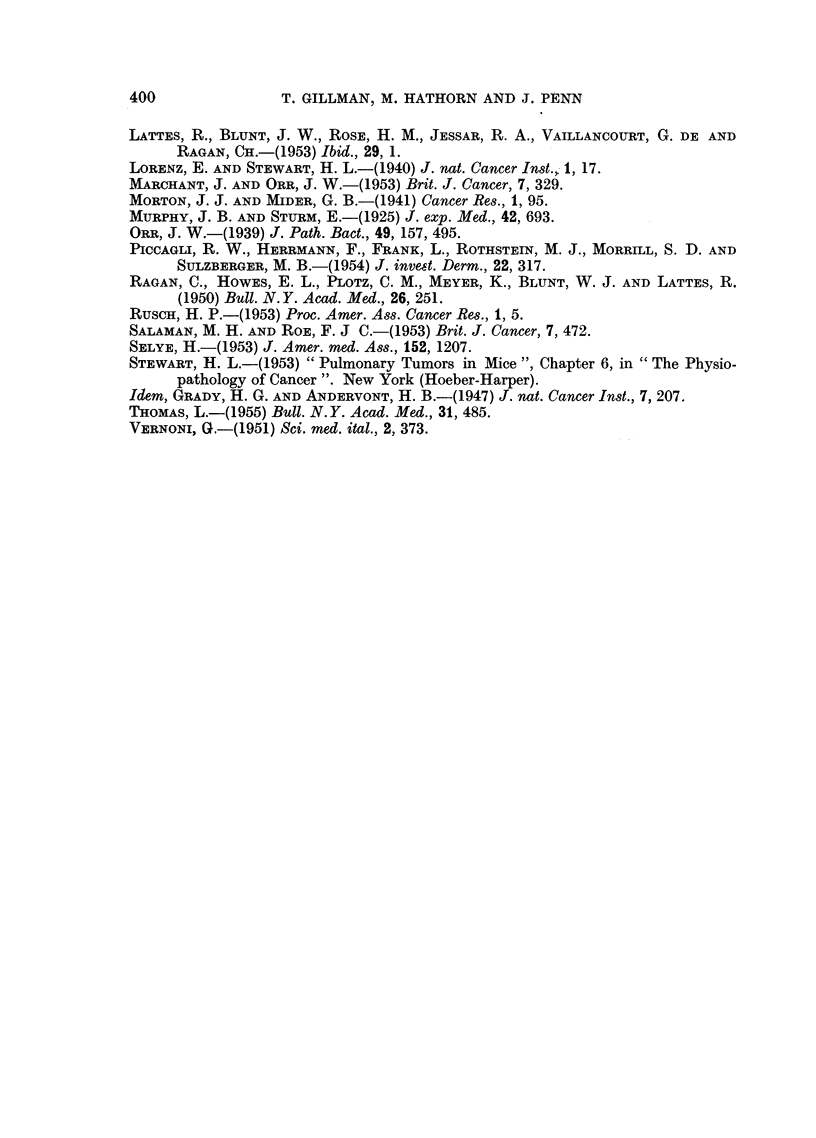# Actions of Cortisone on Cutaneous and Pulmonary Neoplasms Induced in Mice by Cutaneous Applications of Methylcholanthrene

**DOI:** 10.1038/bjc.1956.45

**Published:** 1956-06

**Authors:** T. Gillman, M. Hathorn, J. Penn


### ACTIONS OF CORTISONE ON CUTANEOUS AND PULMONARY NEOPLASMS INDUCED IN MICE BY CUTANEOUS APPLICATIONS OF METHYLCHOLANTHRENE

T. GILLMAN, M. HATHORN AND J. PENN

*From the Department of Physiology and Schlesinger Organisation Medical Research Unit, Faculty of Medicine, University of Natal, Durban, South Africa*

Received for publication February 7, 1956

THE ability of cortisone to expedite the induction, transplantability and metastasis of various types of epidermal and connective-tissue tumours is still somewhat debatable (see Baserga and Shubik, 1954 ; Ghadially and Green, 1954 ; Piccagli *et al.*, 1954, and Gillman *et al.*, 1955 for critical reviews of relevant literature). There is, however, some evidence that cortisone administration may alter somewhat the pathogenesis of methylcholanthrene-induced skin tumours (Boutwell and Rusch, 1953 ; Gillman *et al.*, 1955).

A careful analysis of the literature indicates that discrepancies in the various reported effects of cortisone on the induction, metastasis and transplantability of tumours are largely attributable, firstly, to differences in the times of initiation of cortisone treatment in relation to the application of carcinogens (Ghadially and Green, 1954) and, secondly, to the type of tumour being investigated (see Baserga and Shubik, 1954 for discussion). Thus, Baserga and Shubik who administered cortisone during the " induction " and " developmental " stages of skin carcinomas found that cortisone inhibited tumour development. Ghadially and Green, on the other hand, found that :

> " Local treatment with cortisone, from the commencement of 9 : 10-dimethyl-1 : 2 benzanthracene painting inhibited papillomata formation in the mouse skin, almost completely. Cortisone also produced this effect when allowed to act during the " developmental " phase of carcinogenesis but was quite ineffective when applied solely during the ' pre-induction ' and ' induction ' phases."

We have previously reported that cortisone fails to suppress epithelial growth or neoplasia if given to wounded rabbits or carcinogen-treated mice before, during and after wounding or methylcholanthrene treatment. However, we did find that cortisone delays, although it does not completely inhibit, tumour formation in mice, and also appears to alter somewhat the pathogenesis of the induced skin carcinomas (Gillman, *et al.*, 1955).

It is now generally conceded that cortisone depresses (but does not completely inhibit) connective-tissue growth following wounding or other injuries (Ragan *et al.*, 1950 ; Baker and Whitaker, 1950 ; Selye, 1953 ; Baxter, Schiller and Whiteside, 1951 ; Gillman and Penn, 1956) and suppresses the reactivity of the reticulo-endothelial system, including its ability to manufacture antibodies (see Thomas, 1955, for original findings and review of literature). In our own skin wound-healing experiments in rabbits, we have found that the administration of cortisone, before and during the healing of incised or excised wounds, depresses the regenerative capacity of the connective tissues but does not completely suppress them.

In a previous brief report from this laboratory it was indicated that because of the intimate interdependence of epidermis and dermis during healing and tumour genesis (Gillman *et al.*, 1955 *a*, *b*, *c* ; Gillman and Penn, 1956) the effects of cortisone on tumour induction in the skin, reported by us, may be attributable to the ability of this steroid hormone to depress connective-tissue regeneration without simultaneously interfering with the multiplication of epidermal cells. In the light of this view, the findings reported below are of a special interest for our hypothesis, more especially, as will be indicated, the effects of cortisone treatment on tumour genesis in the skin are somewhat different from its effects on primary lung tumours simultaneously induced by topical application of methylcholanthrene (MCh).

### MATERIAL AND METHODS

In a series of experiments one drop of 0·3 per cent MCh-solution (in acetone) was applied with a dropper twice weekly to the sacral skin of two month-old albino mice for a total of 20 applications (for details see Gillman, Hathorn and Penn, 1956).

In *Experiment 1*, fourteen days after the last MCh application, the mice were divided into four groups, which received the following treatment :

| | |
|---|---|
| A. Cortisone—100 mg. in 1 c.c. 95 per cent alcohol<br>B. Alcohol—1 drop 95 per cent | 1 drop—thrice weekly—to the previously MCh-treated area. |
| C. Cortisone—0·25 mg. in aqueous suspending medium<br>D. Aqueous suspending medium alone, 0·25 c.c. | Thrice weekly subcutaneous injections. |

In *Experiment 2*, thirty-three days after the last MCh application, the mice were divided into three groups which received the following treatments :

| | |
|---|---|
| 1. Cortisone—100 mg. per c.c. in 95 per cent alcohol<br>2. Alcohol—95 per cent | 1 drop—thrice weekly—to previously MCh-treated area. |

3. No treatment after 20th application of MCh (" slow depilators "—see Gillman, Hathorn and Penn, 1956).

In *Experiment 3* mice received initially 20 acetone applications instead of MCh, and thirty-three days later received thrice weekly cortisone applications to the skin as in Experiment 2.

Experiment 1 was terminated at 231 days, and Experiments 2 and 3 at 266 days after the first MCh application. All mice were killed by ether anaesthesia when moribund, or at the conclusion of the experiments. Full post-mortem examinations were conducted and careful notes were made of the macroscopic appearances of the skin and lungs. Paraffin blocks were prepared of the treated sacral skin in all instances, and of the lungs in each case, and other tissues when deemed necessary. Serial sections were frequently made of lesions in the lungs suspected of being neoplasms.

Finally, a group of untreated stock mice of the same age as those in Experiments 2 and 3, were sacrificed when 266 days old, in order to determine the spontaneous incidence of lung tumours in our strain of mice under our experimental conditions.

### RESULTS

### *Skin tumours*

In these experiments it was found that cortisone, administered after completion of MCh treatment, topically in alcohol or by injection in aqueous suspending medium, failed to alter the *microscopically*-determined incidence of skin tumours as compared with the MCh-treated controls receiving alcohol topically or cortisone-free suspending medium by injection. This failure of cortisone to suppress skin tumours was consistently observed whether or not tumours were present at the time cortisone treatment was started. Whether or not the alcohol, used as a solvent for the cortisone, operated as a slight co-carcinogen still remains to be determined. Our findings relating to this aspect of the problem have been outlined elsewhere (Gillman *et al.*, 1955). It may be added also, that, in experiments not hitherto reported in full, cortisone was not found to promote metastasis of macroscopically obvious fungating skin carcinomata, as was apparently the case in the experiments reported by Baserga and Shubik (1954).

### *Lung tumours*

It has long been known that primary pulmonary adenomas can be induced in mice by cutaneous applications of methylcholanthrene or other carcinogens (Murphy and Sturm, 1925 ; Lorenz and Stewart, 1940 ; Morton and Mider, 1941). The mechanism whereby cutaneously applied MCh promotes this type of neoplasm still unknown. Topical application of carcinogens has also recently been shown to expedite urethane-induced pulmonary adenomata (Salaman and Roe, 1953).

A. *Control Groups.*—Twenty-eight mice in all the experiments were found to have tumours of the lung. In two animals, microscopic examination revealed the lung tumours to be squamous epitheliomata, and since these were found in mice bearing large, locally infiltrating skin carcinomas, they were classified as metastases (one each in the topical cortisone and topical alcohol groups).

The lung tumours in the remaining 26 experimental mice, together with those occuring spontaneously in the one untreated stock mouse of the same age as the experimental groups, were regarded as typical primary neoplasms, with microscopic appearances distinct from those of the secondary carcinomas. On the basis of both the macroscopic and microscopic criteria detailed by Grady and Stewart (1940) these tumours were classified as primary pulmonary adenomata.

TABLE I.—*Types of Skin Lesions associated with Primary Lung Tumours*

| Type of skin lesion. | Number of mice with primary lung tumours. |
|---|---|
| Carcinoma | 13 |
| Carcinoma and sarcoma | 2 |
| Sarcoma | 3 |
| Papillomas only | 3 |
| Without skin lesions | 5 |
| Total | 26 |

The condition of the skin of the mice bearing these primary pulmonary tumours is shown in Table I. From this table it will be seen that 5 mice with lung tumours had no detectable skin lesions at all, and that a further 3 mice had only benign skin lesions. This tends to substantiate our opinion that the lung tumours in our mice were indeed primary pulmonary adenomata.

TABLE II.—*Incidence of Primary Lung Neoplasms*

| Experiment No. | Intitial treatment. | Subsequent treatment. | Incidence of primary lung tumours. At 231 days. No. | % | At 266 days No. | % |
|---|---|---|---|---|---|---|
| Stock mice | Nil | Nil | — | — | 1/13 | 8 |
| 3 | Acetone | Cortisone applications | — | — | 0/7 | 0 |
| 2 | 20 bi-weekly applications of methylcholanthrene (0·3% in acetone) | Nil | — | — | 5/10 | 50 |
| 1, 2 | | Alcohol applications | 3/8 | 37 | 8/15 | 53 |
| 1, 2 | | Cortisone applications | 0/7 | 0 | 6/18 | 33 |
| 1 | | Aq. susp. med. injections | 4/7 | 57 | — | — |
| 1 | | Cortisone injections | 0/5 | 0 | — | — |

B. *Effect of cortisone on incidence of primary lung tumours.*—From Table II it will be seen that in the experiments terminated at 231 days, cortisone, whether administered by injection or by topical application appeared to suppress completely the development of primary lung tumours. At this time no tumours were found in any of the 12 cortisone treated mice as compared with 7 out of 15 of the controls.

However, when the findings in Experiment 2 (terminated at 266 days) are considered it is at once apparent that cortisone did not entirely prevent the development of these tumours. However, cortisone did seem significantly to delay their onset to beyond the 231st day of the experiments. The fact that the experiment was not continued for longer than 266 days may alone account for the lower incidence of lung tumours in the cortisone treated mice, even in the second experiment.

Whether or not cortisone, in addition to delaying their onset, would diminish the ultimate incidence of lung tumours if the experiments had been continued for much longer can only be determined by experiments of even longer duration than those reported upon here (see below).

### DISCUSSION

In the present experiments cortisone was initially applied only during the "developmental" phase of carcinogenesis, and well after the "induction phase" had been completed. Our findings in the skins of our mice are, therefore, not in accordance with those reported by Ghadially and Green (1954), nor with those of Baker and Whitaker (1949), Baserga and Shubik (1954) or Engelbreth-Holm and Asboe-Hansen (1953). However, our observations do support those of Rusch (1953) and of Boutwell and Rusch (1953). Nevertheless, it should be carefully noted, since it merits such special attention, that all the investigators who reported that cortisone suppresses experimentally-induced skin neoplasia, conducted their experiments for only relatively short periods, ranging from 21 days (Baker and Whitaker, 1949) to a maximum of 26 weeks (Piccagli *et al.*, 1954), as opposed to the experiments reported on by Boutwell and Rusch, and by ourselves, which extended over very much longer periods (up to 33 and 38 weeks in our own experiments).

As indicated above, there is ample evidence in the literature to demonstrate that although cortisone retards connective-tissue regeneration, healing of wounds is, nevertheless, ultimately satisfactorily achieved even in cortisone-treated subjects (human and animal). We have already noted elsewhere (Gillman *et al.*, 1955) that cortisone suppresses and alters the dermal reactions usually encountered in mice following hair plucking or those associated with the epidermal neoplasia induced by MCh. However, epidermal malignancy ultimately occurred in our mice, albeit the pathogenesis of these tumours was apparently altered by the cortisone treatment administered topically or by subcutaneous injection. These earlier observations from our laboratory are confirmed by the presently reported experiments, in respect of the skin tumours. We may also add that in our mice, both in the present and in several other series of experiments (some reported on in the literature and others not), we have not been able to confirm the finding of Baserga and Shubik (1954) that cortisone treatment promotes metastasis of fungating methylcholanthrene induced skin carcinomas.

Regarding the primary pulmonary tumours which were encountered incidentally in our mice—once more our findings indicate that cortisone delays but does not entirely suppress the development of these tumours. Had we studied only animals killed at 231 days of the experiment then it would certainly have seemed that cortisone treatment had completely suppressed the induction of primary pulmonary tumours by cutaneous applications of MCh. However, the results of a subsequent experiment, of longer duration, indicated that cortisone does not completely suppress primary pulmonary adenomatosis, but rather significantly delays the onset of such tumours, which nevertheless appear in greater numbers than among control completely untreated stock mice of similar ages.

Cortisone has been shown by several investigators, including ourselves, to be incapable of suppressing regeneration of the epidermis during wound healing (Lattes *et al.*, 1953 ; Gillman *et al.*, 1955). However, it is generally agreed (see above) that cortisone delays connective-tissue regeneration in healing or inflammation and is apparently not as inhibitory to the growth of transplanted connective-tissue tumours as to epithelial tumours (Baserga and Shubik, 1954).

Like other investigators (Orr, 1939 ; Marchant and Orr, 1953 ; Vernoni, 1951) we have repeatedly drawn attention to the possible role of the connective tissues in epidermal neoplasia (Gillman *et al.*, 1955). It seems possible, at least, that cortisone delays epithelial tumour formation and growth indirectly via its effects on the associated connective tissues. This may explain the retardation of tumour induction, both in the skin and in the lungs of our MCh-treated mice receiving cortisone.

Another explanation may be suggested to account for the apparently more marked (or more prolonged) retarding action of cortisone on tumour induction in the lung as compared with the skin. As shown by Stewart (1953) metastases of primary pulmonary adenomata often exhibit sarcomatous patterns of growth. Moreover, as finally established by Stewart, Grady and Andervont (1947) these tumours frequently undergo partial or complete sarcomatous transformations on serial transplantation. To the four theories advanced by Stewart (1953) to account for this remarkable change in these apparently glandular epithelial lung tumours, yet another possiblity might be added. This fifth theoretical explanation is that the primary lung tumours arise from cells which are derived, embryologically, from mesenchyme. If this ultimately proves to be the case, then it might be expected, in view of the special suppressive effects of cortisone on other mesenchymal derivatives, that the development of lung tumours would also be markedly inhibited by this hormone. This possibility may also explain the sarcomatous transformation of these tumours on transplantation.

Our findings relating to the effects of cortisone on the skin and lung tumour induction provide a basis for suggesting that more intensive study of the role of connective tissues in the histogenesis of neoplasms is merited. It also seems reasonable to suggest that, by using cortisone, it may yet prove possible to illuminate several aspects of the origins, growth and powers of differentiation of metastases and/or transplants of primary pulmonary adenomas in mice.

### SUMMARY AND CONCLUSIONS

1. The effects of topically applied and of injected cortisone on the induction of skin and primary lung tumours by methylcholanthrene have been examined.

2. Cortisone administered in the present experiments only during the " developmental phase " of carcinogenesis, delayed but did not completely suppress the appearance of skin and lung tumours. The retarding effects of cortisone on tumour induction, following topically applied MCh, was more marked in the case of the lung than the skin.

3. Discrepancies in the literature, relating to the action of cortisone on tumour induction are reviewed and attributed : (*a*) to differences in the time of initiation of cortisone administration relative to treatment with carcinogen, and (*b*) to the fact that in many instances the full effects of cortisone on tumorigenesis could not be assessed because the experiments reported on by others were not conducted for long enough.

4. Comparisons have been drawn between the action of cortisone on epithelium and on connective tissue during wound healing, hair growth and tumorigenesis. The conclusion was drawn that the ability of cortisone to retard tumour induction may be due indirectly to the depressive effects of this hormone on the connective tissues. Some possible implications of this conclusion are discussed for understanding tumorigenesis in general, and in particular, the origin and the peculiarities of the growth of metastases and transplants of primary pulmonary adenomas.

### REFERENCES

BAKER, B. L. AND WHITAKER, W. L.—(1949) *Univ. Hosp. Bull. Mich.*, **15**, 4.—(1950) *Endocrinology*, **46**, 544.

BASERGA, R. AND SHUBIK, P.—(1954) *Cancer Res.*, **14**, 12.

BAXTER, H., SCHILLER, C. AND WHITESIDE, J. H.—(1951) *Plast. reconstr. Surg.*, **7**, 85.

BOUTWELL, R. K. AND RUSCH, H. P.—(1953) *Cancer Res.*, **1**, 9.

ENGELBRETH-HOLM, J. AND ASBOE-HANSEN, G.—(1953) *Acta path. microbiol. scand.* **32**, 560.

GHADIALLY, F. N. AND GREEN, H. N.—(1954) *Brit. J. Cancer*, **8**, 291.

GILLMAN, T., HATHORN, M., AND PENN, J.—(1956) *Ibid.*, **10**, 384.

*Idem* AND PENN, J.—(1956) *Medical Proceedings*, Supplement to Vol. 2, 93–186, March, 1956.

*Idem*, PENN, J., BRONKS, D. AND ROUX, M.—(1955*a*) *Brit. J. Cancer*, **9**, 272.—(1955*b*) *Nature* **176**, 932.—(1955*c*) *Experientia*, **11**, 493.

GRADY, H. G. AND STEWART, H. L.—(1940) *Amer. J. Path.*, **16**, 417.

LATTES, R., BLUNT, J. W., ROSE, H. M., JESSAR, R. A., VAILLANCOURT, G. DE AND RAGAN, CH.—(1953) *Ibid.*, **29**, 1.
LORENZ, E. AND STEWART, H. L.—(1940) *J. nat. Cancer Inst.*, **1**, 17.
MARCHANT, J. AND ORR, J. W.—(1953) *Brit. J. Cancer*, **7**, 329.
MORTON, J. J. AND MIDER, G. B.—(1941) *Cancer Res.*, **1**, 95.
MURPHY, J. B. AND STURM, E.—(1925) *J. exp. Med.*, **42**, 693.
ORR, J. W.—(1939) *J. Path. Bact.*, **49**, 157, 495.
PICCAGLI, R. W., HERRMANN, F., FRANK, L., ROTHSTEIN, M. J., MORRILL, S. D. AND SULZBERGER, M. B.—(1954) *J. invest. Derm.*, **22**, 317.
RAGAN, C., HOWES, E. L., PLOTZ, C. M., MEYER, K., BLUNT, W. J. AND LATTES, R. (1950) *Bull. N.Y. Acad. Med.*, **26**, 251.
RUSCH, H. P.—(1953) *Proc. Amer. Ass. Cancer Res.*, **1**, 5.
SALAMAN, M. H. AND ROE, F. J C.—(1953) *Brit. J. Cancer*, **7**, 472.
SELYE, H.—(1953) *J. Amer. med. Ass.*, **152**, 1207.
STEWART, H. L.—(1953) " Pulmonary Tumors in Mice ", Chapter 6, in " The Physiopathology of Cancer ". New York (Hoeber-Harper).
*Idem*, GRADY, H. G. AND ANDERVONT, H. B.—(1947) *J. nat. Cancer Inst.*, **7**, 207.
THOMAS, L.—(1955) *Bull. N.Y. Acad. Med.*, **31**, 485.
VERNONI, G.—(1951) *Sci. med. ital.*, **2**, 373.